# The Donegal Fund

**Published:** 1889-05-18

**Authors:** 


### THE DONEGAL FUND.

We must leave to posterity the final decision as to whether Mr. O'Brien or Mrs. Ernest Hart has done most for Ireland, but for ourselves we must confess ourselves more in sympathy with the lady than the gentleman. We have before now expressed the opinion that industrial progress does more for the good of the people than political benefits, and the true benefactors of any nation are those who, like Queen Margherita, in Italy, revive and foster the industries of the poor. In the midst of the fighting on both sides--the Coercion Bill and the Plan of Campaign—one or two wise and noble souls have roused the Irish peasantry to energy and independence. Mrs. Ernest Hart was the first to awaken English sympathy in this matter, to bring before the notice of the London public what the Irish peasants could do. The Donegal Industrial Fund, which has now its headquarters at Donegal House, 43, Wigmore-street, W., has given employment both to distressed ladies and others in Ireland, and has taught them new modes of gaining a livelihood. They have proved themselves very apt pupils, and some of the work recently exhibited at Donegal House, where a number of Irish girls have been trained by Miss Una Taylor in art and ecclesiastical embroidery, is exquisite. We may specially mention a set of ecclesiastical vestments ordered by the Duke of Newcastle, in which a beautiful design in passion-flowers is worked on cream-coloured Roman satin, and a Court dress intended for the Countess of Aberdeen. Donegal House is well worthy of a visit from all who care to see how much can be done by personal interest and wise help. Besides, visitors will be able to show their practical sympathy by becoming customers of an establishment where they can buy many useful and beautiful things at remarkably moderate prices.

